# *Rafflesia consueloae* (Rafflesiaceae), the smallest among giants; a new species from Luzon Island, Philippines

**DOI:** 10.3897/phytokeys.61.7295

**Published:** 2016-02-25

**Authors:** John Michael M. Galindon, Perry S. Ong, Edwino S. Fernando

**Affiliations:** 1 Institute of Biology, College of Science, University of the Philippines – Diliman, 1101 Quezon City, Philippines; 2 Diliman Science Research Foundation, Diliman, 1101 Quezon City, Philippines; 3 Department of Forest Biological Sciences, College of Forestry and Natural Resources, University of the Philippines – Los Baños, College, 4031 Laguna, Philippines

**Keywords:** Conservation, ecology, holoparasitic plants, taxonomy

## Abstract

A new species of *Rafflesia* (Rafflesiaceae) from Luzon Island, Philippines, *Rafflesia consueloae* Galindon, Ong & Fernando, is described and illustrated. It is distinct from all other species of *Rafflesia* in its small-sized flowers, the upright perigone lobes, and prominently cream-white disk surface that is often devoid of processes. Its small-sized flowers, with an average diameter of 9.73 cm when fully expanded, make it the smallest of the largest flowers in the world.

## Introduction

**Figure 1.**
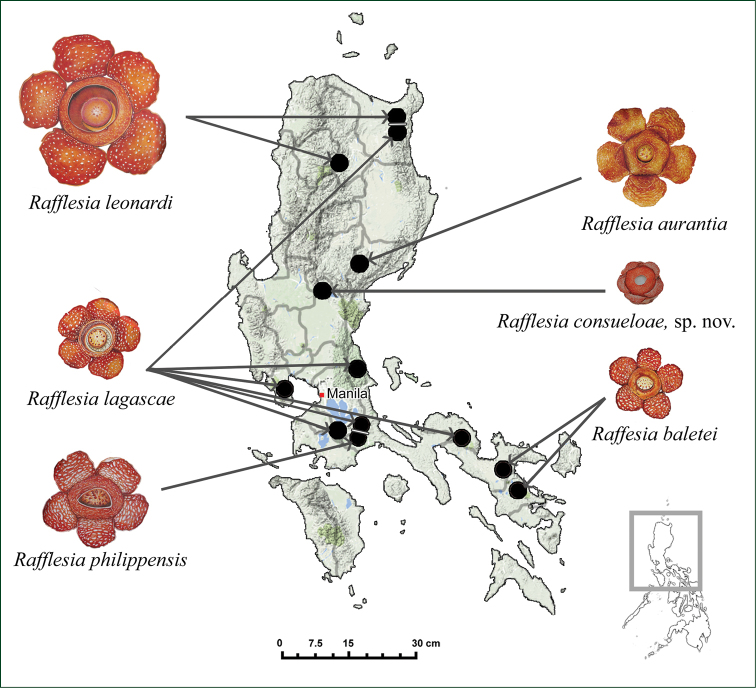
Distribution of the six species of *Rafflesia* on Luzon Island, Philippines, including the new species, *Rafflesia consueloae*. All *Rafflesia* images were drawn by Ms Yasmin S. Ong, five of which were originally published in David et al. (2011). Their use here is with permission of the book publisher, the Energy Development Corporation. All images are scaled relative to the actual sizes of each species. Map source: http://wikimapia.org/#lang=en&lat=12.533115&lon=121.069336&z=6&m=t

*Rafflesia* R.Br. (Rafflesiaceae) is a genus of endophytic, holoparasitic plants, well-known for producing the largest flowers on record (Kuijt 1969, Meijer 1985, 1997, Nais 2001). The Philippines is one of the centers of diversity of the genus (Barcelona et al. 2009b, Pelser et al. 2013), with at least 12 species thus far recorded from the archipelago (Teschemacher 1842, Blanco 1845, Hieronymus 1885, Barcelona and Fernando 2002, Fernando and Ong 2005, Barcelona et al. 2006, 2008a, 2008b, 2009a, 2009b, 2011, 2014, Galang and Madulid 2006, Balete et al. 2010, Pelser et al. 2013), eight of which were described only since 2002. Of all known Philippine species, five are recorded from Luzon Island, viz., *Rafflesia aurantia* Barcelona, Co & Balete (Barcelona et al. 2009a) from Quirino Province; *Rafflesia baletei* Barcelona & Cajano (Barcelona et al. 2006) from Camarines Sur Province; *Rafflesia lagascae* Blanco (Blanco 1845, Barcelona et al. 2009, 2011 [as *Rafflesia manillana* Teschem.], Pelser et al. 2013) from Cagayan, Bataan, Rizal, Laguna, Quezon, and Camarines Norte Provinces; *Rafflesia leonardi* Barcelona & Pelser (Barcelona et al. 2008a, 2011) from Cagayan and Kalinga Provinces, and *Rafflesia philippensis* Blanco (Blanco 1845, Barcelona et al. 2009) [as *Rafflesia banahawensis* Madulid, Villariba & Agoo (2007), and as *Rafflesia banahaw* Barcelona, Pelser & Cajano (2007)] from Laguna and Quezon Provinces (Figure 1).

In this paper, we describe *Rafflesia consueloae*, the 6th species from Luzon Island, and the 13^th^ for the entire Philippine archipelago.

## Taxonomy

### Rafflesia consueloae

Taxon classificationPlantaeMalpighialesRafflesiaceae

Galindon, Ong & Fernando sp. nov.

urn:lsid:ipni.org:names:77153385-1

Figures 2 , 3 , 4

#### Diagnosis.

This species is distinct from all other *Rafflesia* species in its small-sized flowers (average of 9.73 cm diameter), the upright perigone lobes, and the prominently cream-white disk surface which is often devoid of processes. On Luzon Island, it overlaps in the size of mature buds and number of anthers with *Rafflesia baletei* and *Rafflesia aurantia* (Table 1).

**Table 1.** Diagnostic characters separating *Rafflesia consueloae* from *Rafflesia aurantia* and *Rafflesia baletei*. Data on *Rafflesia aurantia* from Barcelona et al. (2009a, 2009b, 2011), *Rafflesia baletei* from Barcelona et al. (2006, 2009b, 2011).

	*Rafflesia consueloae*	*Rafflesia aurantia*	*Rafflesia baletei*
Mature bud diameter (cm)	6.4−9.9 (ave. 8.27 ± 0.82, n=147)	8.5−9	7.5−9
Flower diameter (cm)	6.6–12.7 (ave. 9.73 ± 1.33, n=63)	*c.* 20	9−22
Perigone lobe orientation	generally erect or upright throughout their length; the apex only slightly recurved; the basal parts imbricate	arching, distantly disposed	erect basally, but recurved halfway distally
Perigone color	reddish brown	orange	orange or reddish orange
Diaphragm rim color vs. diaphragm color	whitish when fresh, becoming darker with age	concolorous	darker
Diaphragm surface	warts thin, with blunt whitish tips when fresh, forming variably-shaped impressions of perigone warts	sharp-edged, areoles forming	reticulate
Diaphragm diameter (cm)	3.2–9.0 (ave. 6.87 ± 1.11, n=85)	10	7−8.5 (−12)
Aperture diameter (cm)	1.47–3.85 (ave. 2.57 ± 0.58, n=92)	3−3.6	3−3.5
Number of disk processes	usually absent, or rarely if present centrally disposed	indefinite	19−26
Disk rim	irregularly shallowly to deeply incised	entire	irregularly and shallowly crenulate
Disk surface color	distinctly dull cream white in newly opened flowers	light orangish, with prominent processes	glistening cream-white, becoming reddish brown at the periphery
Disk processes types	when present monomorphic, the tips with brown acicular hairs or bristles	polymorphic, flattened, peripheral ones narrowly lanceolate, spinose	monomorphic, conical, or slightly laterally compressed, often branched
Disk processes maximum length (mm)	3	5–10	10
Ramenta length (mm)	0.5–3, longer towards the base of the tube	7–10	2, longer towards the base of the tube
Number of anthers	12–14	12–14	11–14

#### Type.

PHILIPPINES. Luzon Island: Nueva Ecija Province, Municipality of Pantabangan, Brgy. Fatima, Mt Balukbok, 15°50'17.30"N, 121°05'21.60"E, 325 m elevation, ♂ flowers, 19 March 2014, *Fernando & Galindon 3373* (spirit collection; holotype PNH, isotype PUH).

#### Description.

Endophytic holoparasite. *Mature buds* 6.4−9.9 cm in diameter (average 8.27 ± 0.82 cm, n=147), covered with three overlapping layers of bracts, each layer with five bracts, those in innermost layer up to 6.5 cm long and 5 cm wide, light brown. *Flowers* 6.6–12.7 cm in diameter (average: 9.73 ± 1.33 cm, n=63) when fully expanded, up to *c.* 6.0–13 cm (average 9.49 ± 1.63 cm, n=74) tall. *Perigone lobes* 5, generally upright throughout their length, the apex only slightly recurved, the basal parts imbricate, reddish brown in fresh bloom, becoming darker with age, 3.1–3.8 cm long, 3.2–5 cm wide, orbicular, covered with sharply-edged fine warts and areola-forming ornamentations, the warts dense and powdery white when fresh, concolorous with background tissue with age; the undersurface of topmost lobe generally smooth, others with wart impressions on the distal half; the lobes usually shrinking towards the diaphragm at senescence. *Diaphragm* 3.2–9.0 cm in diameter (average 6.87 ± 1.11 cm, n=85), often slightly darker or rarely concolorous with the perigone lobes, warts thin with blunt whitish tips when fresh, forming variably-shaped impressions of perigone warts; the rim of aperture entire, whitish in fresh bloom, becoming dark with age; diaphragm aperture 1.47–3.85 cm (average 2.57 ± 0.58 cm, n=92); windows absent. *Ramenta* throughout the inner side of the flower tube, glabrous, darker on the tips, denser on the perigone tube floor, each 0.5–3 mm long, slender, apices unbranched, clavate; middle portion 1–2 mm long, cleaved apically up to 3 branches, swollen; sparse towards the aperture, irregular, up to 1.5 mm long. *Disk c.* 4–4.5 cm across, prominently cream-white in newly opened flowers, slightly dome-shaped centrally in male flowers, slightly crateriform in female flowers; the rim prominently raised to slightly arching, to 2.5–3.0 mm high, irregularly serrate and shallowly to deeply incised; brown acicular hairs sparsely scattered all over the disk; disk processes usually absent, or rarely, if present centrally disposed, in male flowers up to 10, in female flowers 11–18, monomorphic, to *c.* 3 mm tall, apex with 2–3 brown bristles. *Column* to 4 cm from the base of the cupule to the upper surface of the disk; neck of column to *c.* 1.5 cm wide; the lower surface of the disk near the rim (corona) concolorous with upper disk surface, in female flowers generally smooth, while in male flowers covered with fine brown bristles, each to *c.* 1 mm long. *Male flowers* with 12−14 anthers, entrenched in sulci 4–7 mm across, the sulci whitish; male flowers occasionally with vestigial ovaries. *Female flowers* with lunate ovary, *c.* 2.9–4 cm wide by 0.6–1 cm tall; female flowers occasionally with vestigial anthers. *Young fruit* 7.2 cm wide × 5 cm tall, top surface coarse, resembling turtle carapace, ovary 5.5 cm wide × 1.5 cm tall, positioned 2.2 cm from the cupule base.

**Figure 2.**
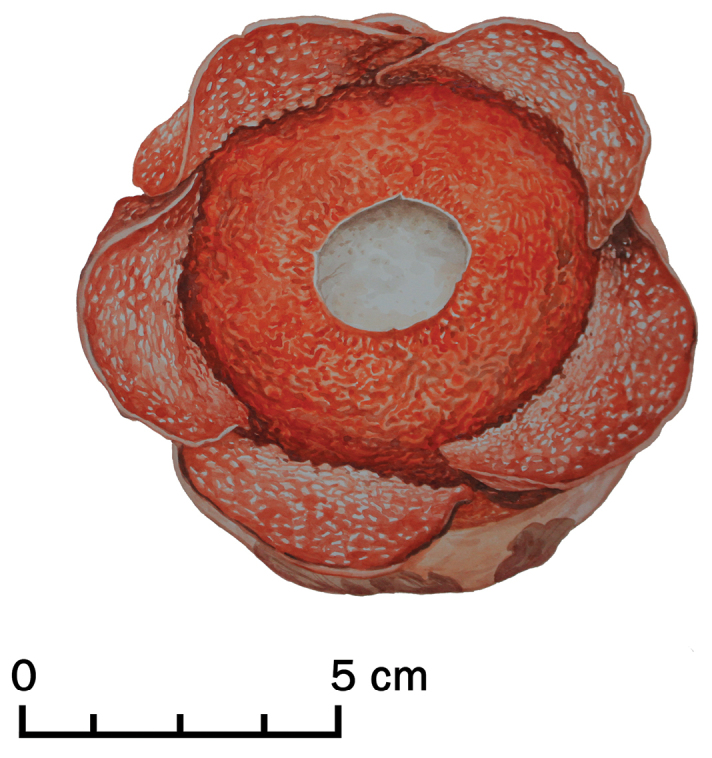
Colour illustration of *Rafflesia consueloae* Galindon, Ong & Fernando based on the holotype, *Fernando & Galindon 3373* (PUH). Colour illustration by Ms Yasmin S. Ong.

**Figure 3.**
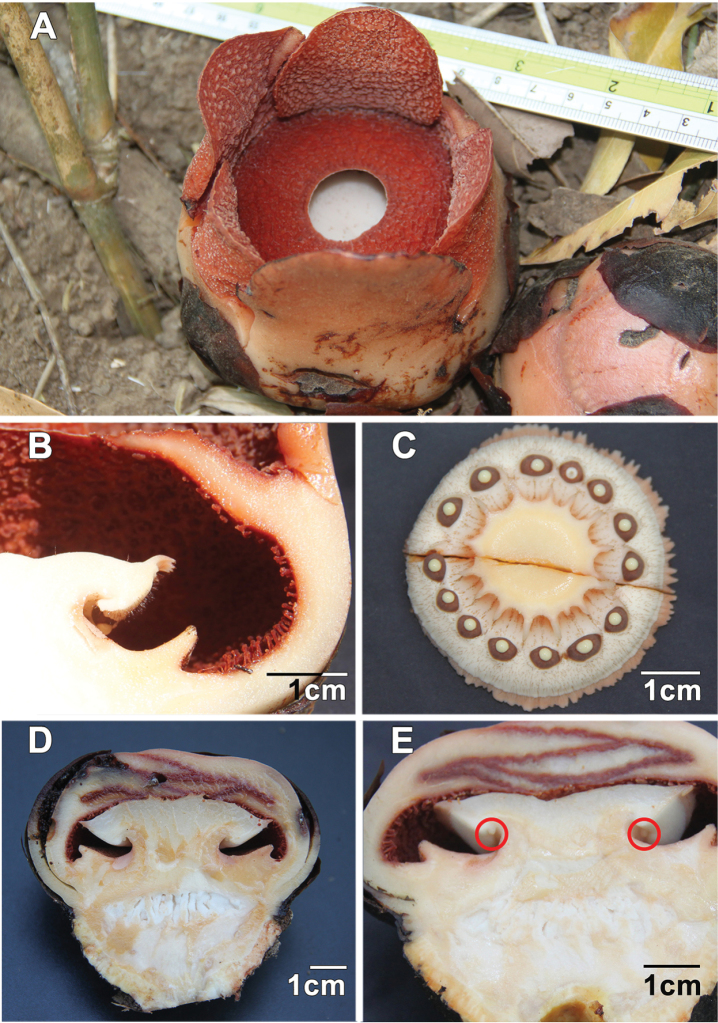
*Rafflesia consueloae* Galindon, Ong & Fernando. **A** Open flower **B** Longitudinal section of flower showing details of ramenta **C** Cross section through column neck showing undersurface of disk with anthers and dense fine bristles **D** Longitudinal section of female bud showing ovary **E** Longitudinal section of female bud showing lower surface of disk with vestigial anthers and generally smooth surface. **A–C** *Fernando & Galindon 3373* **D** *Fernando & Galindon 3378* **E** *Fernando & Galindon 3376*. All photographs by Edwino S. Fernando.

**Figure 4.**
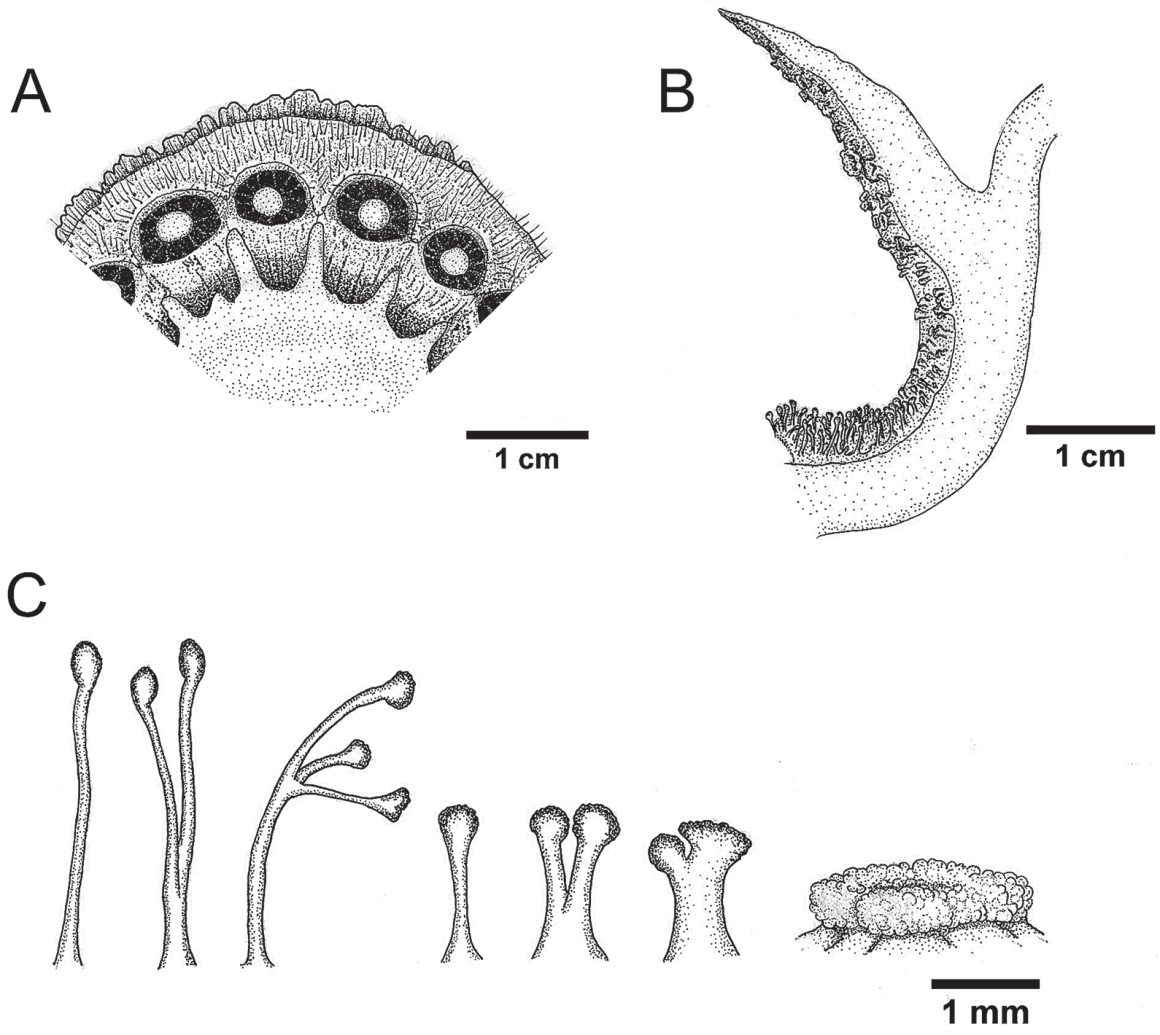
Line drawings of *Rafflesia consueloae* Galindon, Ong, & Fernando. **A** Anthers underneath disk **B** Thin section of perigone tube showing details of ramenta **C** Lower, middle, and upper types (left to right) of ramenta. **A−C** based on *Fernando & Galindon 3373.* Line drawings by Jason B. Fernandez.

#### Distribution.

Endemic to the Philippines. Luzon Island, Nueva Ecija Province, Municipality of Pantabangan. The species is currently known only from two mountain sites with remnants of tropical lowland evergreen rain forests, Mt Balukbok and Mt Pantaburon, about 2 km apart, all within the Pantabangan-Carranglan Watershed.

#### Habitat and ecology.

This species occurs between 300 and 500 m elevation. It is restricted to roots of *Tetrastigma* sp. (Vitaceae) (*Fernando & Galindon 3374*: PUH) growing among climbing bamboo (*Dinochloa luconiae* (Munro) Merr.) thickets. In the type locality on Mt Balukbok, the forest canopy is about 15–20 m tall and includes trees of *Radermachera pinnata* (Blanco) Seem. (Bignoniaceae), *Pterocymbium tinctorium* Merr. (Malvaceae), *Diplodiscus paniculatus* Turcz. (Malvaceae), *Bombax ceiba* L. (Malvaceae), *Maranthes corymbosa* Blume (Chrysobalanaceae), and *Dysoxylum gaudichaudianum* (A.Juss.) Miq. (Meliaceae). The middle canopy consists of *Ficus botryocarpa* Miq., *Ficus ampelas* Burm.f. (Moraceae), *Artocarpus ovatus* Blanco (Moraceae), *Syzygium* sp. (Myrtaceae), *Macaranga tanarius* (L.) Müll.Arg. (Euphorbiaceae), and dense clumps of the erect bamboo *Schizostachyum lumampao* (Blanco) Merr. (Poaceae). On Mt Pantaburon, the populations of *Rafflesia consueloae* are proximate to an old reforestation area planted with *Gmelina arborea* Roxb. (Verbenaceae) and mango (*Mangifera indica* L.; Anacardiaceae).

#### Additional specimens examined.

Philippines. Luzon Island: Nueva Ecija Province, Municipality of Pantabangan, Brgy. Fatima, Mt Balukbok, 15°50'17.30"N, 121°05'21.60"E, 330 m elevation, bisexual flower bud, 19 March 2014, *Fernando & Galindon 3376* (PUH); 15°50'17.30"N, 121°05'21.60"E, 330 m, immature fruit, 19 March 2014, *Fernando & Galindon 3377* (PUH); 15°50'15.19"N, 121°05'21.40"E, 336 m elevation, ♀ flower bud, 19 March 2014, *Fernando & Galindon 3378* (PUH); 15°50'15.19"N, 121°05'21.40"E, 336 m elevation, partially opened ♀ flower, 19 March 2014, *Fernando & Galindon 3379* (PUH); 15°50'12.20"N, 121°05'15.00"E, 380 m elevation, old ♂ flower, 19 March 2014, *Fernando & Galindon 3380* (PUH). Brgy. West Poblacion, Mt Pantaburon, 15°50'36.62"N, 121°05'42.7"E, 435 m, ♂ flower, 14 February 2015, *Fernando & Galindon 3667* (PUH, PNH); 15°50'36.62"N, 121°05'42.7"E, 435 m, ♂ flower, 16 May 2015, *Fernando & Galindon 3773* (PUH); 15°50'37.8"N, 121°05'44.9"E, 437 m, ♂ flower, 16 May 2015, *Fernando & Galindon 3774* (PNH).

#### Etymology.

The specific epithet honors Ms Consuelo ‘Connie’ Rufino Lopez, lifelong partner of industrialist Oscar M. Lopez, and a plant lover in her own right. Both delight in culturing, growing and tending their garden which includes more than 100 species of trees, orchids and other plants. With her demure but strong personality, traits which *Rafflesia consueloae* possess, she provides the inspiration for Mr Lopez’s pursuit of biodiversity conservation in the Philippines.

#### Notes.

Prior to this discovery of *Rafflesia consueloae*, *Rafflesia baletei* (Barcelona et al. 2006; David et al. 2011), with flowers (9–) 15–16 (–22) cm in diameter when fully expanded, held the record of being the smallest *Rafflesia*. Our new species, *Rafflesia consueloae*,﻿ has flowers with an average diameter of only 9.73 ± 1.33 cm (range 6.6–12.7 cm; n=63) when fully expanded, making it the smallest of the largest flowers in the world. The disk surface of *Rafflesia consueloae* is also distinctly cream-white in newly opened flowers and is almost always devoid of processes. This character is reminiscent of *Rafflesia rochussenii* Teisjm. & Binn. (Teijsmann 1850) from Java and Sumatra in Indonesia (Meijer 1997, Nais 2001). The absence of processes on the disk is also known in young flowers of *Rafflesia leonardi* (Barcelona et al. 2008a), but the disk in this species is larger (7–8 cm in diameter) and described as ‘tan centrally, purplish towards the periphery’. *Rafflesia consueloae* is the third *Rafflesia* species in the Philippines reported with bisexual flowers. The other two are *Rafflesia baletei* from southeastern Luzon (Barcelona et al. 2006) and *Rafflesia verrucosa* from eastern Mindanao (Balete et al. 2010). In *Rafflesia baletei*, male and female male flowers were described separately to have vestigial organs of the other sex (Barcelona et al. 2006, 2009). But, Barcelona et al. (2009, 2011) also described the flowers of this same species as bisexual. *Rafflesia verrucosa* is also recorded to have bisexual flowers (Balete et al. 2010), although male and female flowers were not described separately. Thus, *Rafflesia consueloae* can also be considered to have bisexual flowers. Whether the flowers are functionally bisexual still has to be shown. Referring to *Rafflesia baletei* and *Rafflesia verrucosa*, Balete et al. (2010) states that ‘It remains to be demonstrated however, whether either or both species are functionally bisexual.’ Further studies on these three species, *Rafflesia baletei*, *Rafflesia verrucosa*,﻿ and *Rafflesia consueloae*, should help provide further insights on this issue.

#### Conservation status.

Following the IUCN Categories and Criteria (IUCN 2012), we regard this species as Critically Endangered (CR B1+2bc). The extent of occurrence of the two small populations of *Rafflesia consueloae* is less than 100 km^2^. Both populations are under the jurisdictional control of the National Irrigation Administration (NIA) and the Pantabangan-Carranglan Watershed Protected Area Management Board. The First Gen Hydro Power Corporation operates the Pantabangan hydroelectric facilities in the area and helps provide support in monitoring the surrounding forests and its biodiversity. However, the continued protection of the *Rafflesia consueloae* populations and other biodiversity in the area needs to be ensured as some local people still hunt wildlife there and forest fires are likely in the dry season. The vertebrate wildlife may also play a role in the biology of the *Rafflesia consueloae*.

How new *Tetrastigma* hosts get infected with *Rafflesia* seeds remains unknown. Several species of wildlife such as tree shrews, rodents, squirrels, wild pigs, elephants, and even ants have been suggested as potential seed dispersers of *Rafflesia* (Emmons et al. 1991, Hidayati et al. 2000, Nais 2001, Pelser et al. 2013); that these wildlife species might play an important role in the completion of the Rafflesia’s life cycle through the infection of new *Tetrastigma* hosts, had long been suspected but remains unproven. However, using motion-activated camera traps set up around fruits of *Rafflesia consueloae*, we were able to photograph at least two species of rodents feeding on different occasions (unpublished data, this study). What role these rodents and other wildlife species play in the life cycle of *Rafflesia consueloae* is subject of further study. The current two sites are known hunting grounds of wildlife by some members of the local community. Given the restricted range of this new species, hunting of wildlife might further exacerbate its fragile existence. Also, hunting might increase the chances of forest fires occurring, which are likely in the dry season based on personal observations and interviews with locals.

## Supplementary Material

XML Treatment for Rafflesia consueloae
